# Gelatin and Carboxymethyl Chitosan Edible Coating Incorporated with Carvacrol: Development and Application in Strawberries

**DOI:** 10.3390/foods14193297

**Published:** 2025-09-23

**Authors:** Anthony Pius Bassey, Chaoxiong Meng, Yin Zhang, Fan Wang, Mustapha Muhammad Nasiru, Han Wu, Isaiah Henry Ibeogu, Linlin Fan, Xiaoli Liu

**Affiliations:** 1 Institute of Agro-Product Processing, Jiangsu Academy of Agricultural Sciences, Nanjing 210014, China; bassey_ap44@outlook.com (A.P.B.); 2222318103@stmail.ujs.edu.cn (C.M.); 2023808103@stu.njau.edu.cn (Y.Z.); wangfan713@126.com (F.W.); mustaphamnasiru@gmail.com (M.M.N.); hanwu12065@163.com (H.W.); fanlinlin@jaas.ac.cn (L.F.); 2 State Key Laboratory of Meat Quality Control and Cultured Meat Development, MOST: Key Laboratory of Meat Processing, MARA: College of Food Science and Technology, Nanjing Agricultural University, Nanjing 210095, China; ibeoguhenry@gmail.com; 3 Zhejiang Key Laboratory of Agri-Food Resources and High-Value Utilization, Hangzhou 310013, China

**Keywords:** strawberries, edible coating, active packaging, postharvest quality, preservation

## Abstract

This study developed antimicrobial composite coatings from gelatin (GL) and carboxymethyl chitosan (CMCS) functionalized with carvacrol (CA) as a natural alternative for the preservation of strawberries. Films incorporated with 0%, 1.5%, and 3% CA were characterized by their physical, mechanical, and structural properties. The addition of CA significantly improved opacity, tensile strength, elongation-at-break, and thickness, while reducing water vapor permeability, moisture content, and solubility (*p* < 0.05). Spectroscopy and microscopy also confirmed CA’s uniform dispersion within the matrix. During 14 days of storage at 4 °C, strawberries coated with GL/CMCS/CA-3% significantly reduced weight loss (50.91%) and fungal contamination, improved firmness (79.31%), and maintained color and pH. Conversely, uncoated strawberries showed rapid declines in soluble solids, acidity, phenolics, anthocyanins, vitamin C, and antioxidant activity (*p* < 0.05). Microbial growth was effectively suppressed in coated fruits, while sensory profiles were drastically impaired in uncoated samples at the end of storage (*p* < 0.05). These results demonstrate that GL/CMCS/CA coatings can effectively preserve strawberry quality and extend shelf life without synthetic preservatives.

## 1. Introduction

Strawberries (*Fragaria x ananassa* Duch.) are a popular fruit consumed worldwide. Their appeal comes from their delicious taste and aroma, as well as their high nutritional value, which includes vitamins, minerals, anthocyanins, and other phenolic compounds [1]. Fresh strawberries are commercialized in almost 80 countries, with a total estimate of $19.54 billion in 2023 and projected to reach $24.45 billion by 2030 [2]. Nonetheless, their high water content and dense nutrient composition make them susceptible to microbial contamination, mechanical and physiological lesions, and post-harvest defects, impeding their shelf life. Numerous preservation methods have been employed to tackle these constraints, such as cold storage, chemical disinfection, modified atmospheres, and bioactive packaging. Proper packaging has been exceptional in slowing down deteriorative actions and extending the storage period of foods.

Recent research highlights edible coatings as a promising, natural, and economical alternative for strawberry preservation [3,4,5,6]. Materials rich in carbohydrates, proteins, and lipids are suitable for use as they form a semi-permeable membrane on the fruit’s surface, insulating against microorganisms, gases, and water vapor [3,7]. For example, Gelatin (GL), a hydrophilic derivative protein formed by alkali or acid hydrolysis of collagen from animal tissue, exhibits good biocompatibility/biodegradability, water vapor barrier, and emulsifying characteristics. However, its antioxidant activity is impaired if incorporated individually in food delivery systems [8]. Also, carboxymethyl chitosan (CMCS), derived from modifying the hydroxyl and free amino groups of chitosan, has been applied in films, capsules, and coatings because of its remarkable antimicrobial and antioxidant impacts against oxidative inducers and predominant spoilers in foods. Although several studies have posited that the composite membranes obtained from GL and CMCS demonstrated better physico-mechanical properties than single applications [9,10,11,12], their hydrophilicity gradually disrupts their barrier and water resistance characteristics. Moreover, their molecular structures contain numerous polar groups (-OH, -NH_2_, -COOH) that form hydrogen bonds with water molecules, making films or coatings produced from them prone to moisture. Films may swell, plasticize, or lose barrier properties under refrigeration conditions. Hence, applying essential hydrophobic substances could augment these limitations, including antimicrobial functionality.

Carvacrol (CA) is a phenolic monoterpenoid (5-isopropyl-2-methylphenol) obtained from various plant sources, such as thyme, oregano, pepperwort, and wild bergamot [13]. The hydrophobic molecules may physically disrupt the continuous network of hydrophilic polymer chains, thereby reducing the overall ability of the film matrix to interact with and absorb water molecules [14]. Also, the dispersed hydrophobic droplets within the material create a longer and more winding path for water vapor and gas molecules to diffuse through. CA can partition into and disrupt the lipid bilayer of microbial cell membranes, cause leakage of cellular contents (ions, ATP), and interfere with cellular enzymes and functional proteins, leading to cell death. For example, chitosan bio-nanocomposite coatings loaded with cellulose nanocrystal and carvacrol enhanced the barrier action against O_2_ and moisture, suppressing the respiration and ethylene production of the coated fruits (banana and mango), thereby prolonging their shelf life [15]. In addition, the polymer matrix (GL/CMCS) can act as a carrier for these antimicrobials, potentially controlling their release rate to the fruit surface, providing prolonged protection throughout the storage period. These effects have triggered researchers to integrate CA with GL and/or CMCS in developing edible coatings for food packaging [14,16,17].

Although CA’s antimicrobial potency in films has been extensively studied in various foods, such as meats [18,19], dairy [20], seafood [8,21,22], and fruits [23,24,25], there is limited literature on its effect on edible coating, either via spray or immersion. Thus, this study developed GL/CMCS/CA-based antimicrobial coatings for preserving the quality and extending the shelf life of strawberries. Composite films were produced for physical, mechanical, and structural characterization, while physicochemical, biochemical, and microbiological analyses were performed on coated strawberries stored for 14 days at 4 °C to provide the scientific baseline for food industries on innovative strategies to improve the preservation of fruits, not limited to strawberries, through edible coatings.

## 2. Materials and Methods

### 2.1. Materials

Gelatin (GL), with CAS No. 9000-70-8, jelly strength of 209 Bloom, and a particle size of 8 mesh, was supplied by Sinopharm Chemical Reagent Co., Ltd. (Ningbo, China). Carvacrol (CA, 99% purity, CAS No. 499-75-2, molecular weight (MW) = 150.22 kDa) and carboxymethyl chitosan (CMCS, CAS No. 83512-85-0, MW = 240 kDa, degree of deacetylation = >90%, degree of substitution = 90%) were obtained from Macklin Biochemical Co., Ltd. (Shanghai, China). Glycerol (CAS No. 56-81-5, MW = 92.09 kDa) was purchased from Jiangsu Qiangsheng Functional Chemistry Co., Ltd. (Changshu, China). Anhydrous oxalic acid and acetic acid were sourced from Shanghai Aladdin Biochemical Technology Co., Ltd. and Shanghai McLean Biochemical Technology Co., Ltd. (Shanghai, China), respectively. All other chemicals and reagents were of analytical grade and used without further purification.

### 2.2. Film Development

To assess the effect of incorporating GL, CMCS, and CA to form GL/CMCS/CA films, 2 g of GL (2% *w*/*v*) and 1 g of CMCS (1% *w*/*v*) were added to distilled water (100 mL). The mixtures were heated to 60 °C and continuously stirred at 800 rpm for 2 h to ensure complete dissolution. Thereafter, 3 mL of glycerol was added to plasticize the solution and rested for 30 min. The temperature was then reduced to 40 °C before CA was added at 0%, 1.5% and 3% concentrations (*w*/*w* of total GL/CMCS solids) to derive GL/CMCS/CA-0%, GL/CMCS/CA-1.5%, and GL/CMCS/CA-3% and stirred (90 min) to ensure homogeneous distribution. The pH/viscosity of the emulsion was measured (ZD-2 benchtop pH meter, INESA Scientific Instrument, Shanghai, China) and noted as 5.71/64 mV, 5.98/54 mV, and 6.10/45 mV, respectively. After pouring 25 mL of emulsion into Petri dishes (90 × 15 mm), films were formed by oven-drying at 60 °C. Subsequently, all films were conditioned at 25 °C and 50% relative humidity (RH) for 18–24 h before analysis.

### 2.3. Physico-Mechanical Characteristics of the GL/CMCS/CA Films

#### 2.3.1. Opacity and Water Vapor Permeability (WVP)

Film opacity was determined using a UV-722 spectrophotometer (Shanghai Lengguang Technology Co., Shanghai, China). Film samples (80 × 50 mm) were placed directly against the internal wall of a spectrophotometer cuvette, and the absorbance at 600 nm was measured. The test was performed in triplicate. The final value was calculated using the formula:

(1) $$Opacity=\;\frac{Abs600}{T}\times 100$$

where *T* = film thickness (mm)

The protocol of our previous study was employed for WVP measurement [8]. Briefly, the films were cut (80 × 80 mm) and placed in a desiccator consisting of saturated potassium sulfate. The desiccator was maintained at 25 °C and 75% relative humidity (RH). Weight changes were measured after 12 h and recorded.

#### 2.3.2. Tensile Strength (TS) and Elongation-at-Break (EB)

TS and EB of the films were determined using a texture analyzer (CT3, Brookfield Engineering Labs, Abington, PA, USA). Film strips (80 × 10 mm) were mounted between the tensile grips with an initial gauge length of 50 mm and stretched at a crosshead speed of 50 mm/min until fracture. A 5 kN load cell was used to measure the force. The test was carried out in triplicate, and the values were calculated using the following:

(2) $$TS\left(Mpa\right)=\;\frac{F}{T\times W}\;\times 100$$

(3) $$EB\left(\%\right)=\frac{∆L}{L}\times 100$$

where *F* = extension resistance (N), *T* = sample thickness (mm), *W* = sample width (mm), *L* = initial length between sample grips (mm), ∆*L* = distance increase at which the sample breaks (mm).

#### 2.3.3. Moisture Content (MC), Swelling Degree (SD), and Water Solubility (WS)

These parameters were measured as described by Ibeogu et al. [26] with a slight change. Briefly, films (2 × 2 cm) were weighed (W_0_) and dried for 3 h. The dried samples were weighed (W_1_), immersed in 50 mL of deionized water, sealed, and maintained at 25 °C for 12 h. Afterwards, each sample was carefully removed and weighed (W_2_), surface water was gently blotted away with filter sheets, and the swollen mass was immediately recorded (W_3_). Every test was carried out in triplicate, and the properties were calculated as follows:

(4) $$MC\left(\%\right)=\frac{W0-W1}{W0}\;\times 100$$

(5) $$SD\left(\%\right)=\frac{W2-W0}{W0}\times 100$$

(6) $$WS\left(\%\right)=\frac{W1-W3}{W1}\times 100$$

#### 2.3.4. Color and Thickness

Film color was measured using a Chroma Meter (CR-400, Minolta, Osaka, Japan) calibrated with a standard white tile (Calibration Plate CR-A43). Using a D65 illuminant and a 10° viewing angle, the CIE L* (lightness), a* (red-green), and b* (blue-yellow), ΔE* (total color difference) were recorded from four random locations per sample.

A potable Vernier caliper (Fuzhou Winwin Industrial Co., Ltd., Fuzhou, China) with a diameter of 0–150 mm was used to measure the thickness of the films. This was achieved in five different areas, and the average was recorded.

### 2.4. Film Characterization

#### 2.4.1. Fourier Transform Infrared (FT-IR) Spectroscopy

The chemical structures of the raw materials (GL, CMCS, CA) and the composite films were analyzed using Fourier Transform Infrared spectroscopy (Vertex 80, Bruker Optik GmbH, Ettlingen, Germany). Spectra were acquired in the range of 500–4000 cm^−1^ with a resolution of 4 cm^−1^, accumulating 32 scans per spectrum to enhance the signal-to-noise ratio. The analysis was carried out in triplicate.

#### 2.4.2. Scanning Electron Microscopy (SEM)

The surface and cross-sectional morphology of the films were examined by a scanning electron microscope (Hitachi S-3000N, Tokyo, Japan). Film samples (4 × 4 mm) were mounted on aluminum stubs using conductive tape. Before imaging, the samples were sputter-coated with a thin layer of gold to ensure electrical conductivity. Micrographs were obtained at an accelerating voltage of 10 kV. The analysis was carried out in triplicate.

#### 2.4.3. X-Ray Diffraction (XRD) Analysis

The crystallinity of the films was analyzed following the protocol in our previous study [8]. XRD patterns were recorded using an Empyrean diffractometer (Malvern Panalytical Ltd., Grovewood Road, Malvern, UK) with Cu-Kα radiation (λ = 1.54 Å) generated at 30 kV and 10 mA. Samples (1 × 1 cm) were scanned over a 2θ range of 5 to 80° C at a scanning speed of 15.6°/min. The analysis was performed in triplicate.

### 2.5. Application of GL/CMCS/CA Coatings on Strawberries

The preservation effectiveness of the coatings was investigated by measuring the physicochemical, biochemical, and microbiological indices on fresh strawberries. Strawberries (cultivar Camino Real) were obtained from a reputable fruit vendor in Nanjing and transported immediately to the Institute of Agro-product Processing laboratory at the Jiangsu Academy of Agricultural Sciences (Nanjing, China). The fruits were brought directly from the field and pre-selected based on size and ripeness. The selected fruits (average size = 25 ± 0.3 g) were sanitized in a 2% (*v*/*v*) sodium hypochlorite solution for 30 s, rinsed, and air-dried for 2 h. Following the optimization of the films after characterization, the fruits were randomly divided into three groups: Control/uncoated = CON, GL/CC/CA-1.5% = CA-1.5%, and GL/CC/CA-3% = CA-3%. The coating solution was prepared according to Section 2.2 without oven-drying (film formation). The samples (five strawberries per group) were suspended by their calyx to ensure an even air circulation and placed in a controlled chamber (25 °C, 40% RH) for 90 min before being placed in plastic boxes, weighed (125 ± 0.2 g), and refrigerated (4 °C) for 14 days. The experimental parameters were measured at five storage periods (0, 4, 7, 10, and 14 d).

### 2.6. Physicochemical Analyses of the Strawberries

#### 2.6.1. Weight Loss (WL) and Decay Rate

The standard method of the Association of Official Analytical Chemists [27] was followed to determine the WL of the strawberries at each storage period. The difference between measurements was calculated according to Equation (1) and expressed as the percentage WL of each treatment. The measurement was performed in triplicate.

(7) $$WL\;\left(\%\right)=\frac{W0-W1}{W0}\times 100\;$$

where W_0_ = weight on day 0 of each sample and W_1_ = weight on the subsequent storage period of each sample.

For decay rate, the fruits were visually assessed for the presence and/or fungi growth, including brown spotting or bacterial lesions, during storage. The measurement was performed in triplicate, and the % contamination was calculated using the equation below:

(8) $$Decay\;rate\left(\%\right)=\;\frac{contaminated\;fruits}{total\;fruits}\times 100\;$$

#### 2.6.2. Firmness and pH

A CT3-type TA.XTplus Texture Analyzer (Stable Micro Systems Ltd., Surrey, UK), equipped with a 5 N load cell, was used to measure the firmness of the strawberries. The device was set as follows: diameter probe = 4 mm, speed = 1 mm s^−1^, penetration distance = 2 mm, point load = 3 g (0.03 N), and contact area = 12 mm^2^. Three repetitions were made in the distal region (softest part) of the fruits (n = 5). The measurement was performed in triplicate, and the results were expressed in Newton (N) [28].

For pH, the strawberries were liquefied at each storage interval and recorded using a benchtop pH meter (ZD-2, INESA Scientific Instrument, Shanghai, China). The measurement was performed in triplicate.

#### 2.6.3. Color

The method described in Section 2.3.4 was followed for color measurement of the samples. The total color difference (ΔE) was calculated using Equation (9), where *t* signified the storage periods and 0 signifies the initial period. The measurement was performed in triplicate.

(9) $$\Delta E=\sqrt{{\left(L_{t}^{*}-L_{0}^{*}\right)}^{2}+{\left(a_{t}^{*}-a_{0}^{*}\right)}^{2}+{\left(b_{t}^{*}-b_{0}^{*}\right)}^{2}}$$

### 2.7. Biochemical and Antioxidant Activities of Coated Strawberries

#### 2.7.1. Total Soluble Solids (TSS) and Titratable Acidity (TA)

A digital refractometer (HI96813, HANNA Instruments, Bedfordshire, UK) was used for TSS measurement. A few drops of the sample filtrate were placed on the device’s prism glass, and the result was expressed in °Brix.

For TA, the sample (5 g)was homogenized in distilled water (50 mL), filtered, and 1 mL filtrate was titrated with 0.1 M NaOH to a pH = 8 endpoint [5]. The volume of NaOH required to achieve the endpoint was noted per sample. The analyses were performed in triplicate, and the results were calculated as follows:

(10) $$TA\;\left(\%\;citric\;acid\right)=\frac{Vol.NaOH\;\times 0.1\;\times 0.064}{5}$$

#### 2.7.2. Total Phenolic Content (TPC)

The method outlined by Bertolo et al. was employed to analyze the TPC of the strawberries with minor changes [3]. A sample aliquot (5 mL) was diluted in 40 mL of 50% methanol and centrifuged (4000× *g*, 20 °C, 15 min). From the supernatant, 50 μL was combined with 50 μL of Folin–Ciocalteu reagent. After 5 min, 200 μL of 7% (*w*/*v*) sodium carbonate was added to alter the reaction. After 15 min incubation, absorbance was read at 725 nm in a P5 UV/Vis spectrophotometer (Shanghai Measuretech Instrument Co. Ltd., Shanghai, China). A gallic acid standard curve (20–500 μg/mL) was used for quantification, and results were expressed as mg GAE/g extract. The analysis was conducted in triplicate.

#### 2.7.3. Total Anthocyanin Content (TAC)

The pH differential method was used to quantify the TAC of the samples [29]. The supernatant from Section 2.4.1 was diluted in buffer solutions = 1.0 (0.025 mol L^−1^ HCl and KCl) and 4.5 (0.4 mol L^−1^ sodium acetate and acetic acid), in a 1:9 ratio. After 15 min incubation, the absorbances were read at 510 and 700 nm. The test was performed in triplicate, and the content was calculated using the formula:

(11) $$TAC\;(mg/L)=\frac{\left(A1.0-A4.5\right)\;\times 449.2\;\times DF\;\times 1000}{26900\;\times L}$$

where A_1.0_ = absorbance at pH 1.0, A_4.5_ = absorbance at pH 4.5, 449.2 = molecular weight (g/mol) of cyanidin-3-glucoside (the most common anthocyanin), DF = dilution factor, 26,900 = molar extinction coefficient (ϵ) of cyanidin-3-glucoside (L/mol·cm), L = path length of the cuvette (usually 1 cm), and 1000 = conversion factor from g to mg.

#### 2.7.4. Vitamin C Content

Vitamin C content was determined by titration with 2,6-dichlorophenolindophenol (DCPIP) according to the Tillmans method [29]. A 5 mL sample was added to 25 mL of 0.5% oxalic acid (*w*/*w*) and centrifuged (3500× *g*, 15 min, 25 °C). Afterwards, 5 mL supernatant was titrated with a 0.1% (*w*/*w*) DFI solution while stirring continuously until the solution turned a light pink color (endpoint of the titration) that persisted for about 15 s. The test was performed in triplicate and reported as mg ascorbic acid per 100 g of strawberries.

#### 2.7.5. Antioxidant Activity

The DPPH protocol detailed in our previous study was applied to determine the antioxidant activity of the coated strawberries [8]. Briefly, homogenized sample (50 μL) was reacted with 200 μL of DPPH• methanolic solution (0.2 mM) and left in the dark for 30 min before absorbance measurement at 517 nm. The test was performed in triplicate, and the result was calculated using the formula:

(12) $$Antioxidant\;activity\;(\%\;DPPH)=1-\frac{A_{1}-A_{2}}{A_{0}}\times 100$$

where A_0_ = initial absorbance; A_1_ = sample absorbance; A_2_ = sample with methanol.

### 2.8. Microbiological Enumeration

Microbiological enumeration of total viable counts (TVC) and fungal counts (FC) was performed based on the method in a previous study [30]. Briefly, a 5 mL sample was mixed with 45 mL of sterile saline water (0.85% NaCl) and homogenized for 10 min. The homogenate was serially diluted (1:10), and 100 µL diluent was spread on plate count agar (PCA; Qingdao Hopebio-Technology, Qingdao, China) and potato dextrose agar (PDA; Qingdao Hopebio-Technology, China) for TVC and FC, respectively. The plates for TVC were incubated at 37 °C for 48 h, and those for FC at 28 °C for 72 h before colony counting. The result was achieved in triplicate and expressed as log CFU/g.

### 2.9. Sensory Evaluation

The procedure of Hassani et al. [31] was adopted for the sensory evaluation with minor changes. The panelists were graduate students of the Institute of Agro-products Processing, Jiangsu Academy of Agricultural Sciences (Nanjing, China) and consisted of 12 evaluators (seven females and five males; average age of 21 years). They were non-smokers without any underlying olfactory dysfunction before evaluation. Strawberries were evaluated at different storage intervals in a controlled, well-lit, and odor-free environment. The samples were randomly coded, and the purpose of the assessment was not shared with panelists. The evaluation comprised appearance, taste, firmness, and overall acceptability, and was measured using a 9-point hedonic scale (where 1 = extremely dislike and 9 = extremely like). To prevent carry-over effects, panelists rinsed their palates with water between sample evaluations.

### 2.10. Statistical Analysis

Data analysis was performed with IBM SPSS v.25 software (IBM Corp., Armonk, NY, USA). A two-way ANOVA was selected over a one-way ANOVA to enable the simultaneous analysis of the main effects of the two independent factors (group and storage period) and their potential interaction on each dependent variable. For any significant main or interaction effects (*p* < 0.05), means were separated using Tukey’s HSD post hoc test. All data were expressed as mean values ± standard deviation. Data visualization and graph plotting were accomplished using OriginPro 2024 software (OriginLab Corp., Northampton, MA, USA).

## 3. Results

### 3.1. Physical and Mechanical Characteristics of the GL/CMCS/CA-Based Films

#### 3.1.1. Opacity and WVP

Given the critical importance of optical transparency and barrier characteristics in food packaging [32], the opacity and WVP of the fabricated membranes were analyzed (Table 1). The result ranged from 1.41 to 3.62 mm, with the highest value observed in GL/CMCS/CA-3% films compared to the Control, which was slightly lower than the value (3.88 mm) in our previous study [8]. This indicates a positive correlation between the optical property of the films and CA concentration. Although high visual transparency allows consumers to inspect the product, it also ensures adequate UV protection against photo-oxidation and degradation of the contents [33].

WVP describes a membrane’s barrier performance against moisture, a key property for food packaging, where a low WVP is essential to extend shelf life. Its value is primarily governed by three factors: the intrinsic properties of the biopolymers used, membrane microstructure, and environmental conditions [34]. As shown in Table 1, the WVP of the films decreased from 4.07 (GL/CMCS/CA-0%) to 2.51 × 10^−11^ g^−1^ s^−1^ Pa^−1^ (GL/CMCS/CA-3%), as CA increased (*p* < 0.05), which may be linked to the denser structure created by the interaction between GL/CMCS polymers and CA. Shihao et al. [35] posited that during fabrication, hydrophilic groups can form intermolecular hydrogen bonds among themselves, facilitating a structural reorganization of the matrix, leading to a reduced pore space and the formation of a denser, more compact film morphology. This refined architecture effectively obstructs the diffusion pathway for water molecules, thereby improving the overall barrier performance.

#### 3.1.2. TS and EB

The TS and EB of the GL/CMCS/CA films are presented in Table 1. The addition of CA in the polymer matrix significantly enhanced the TS of the films, with GL/CMCS/CA-3% exhibiting the highest values (42.70 MPa). This was higher than the 27.10 MPa reported for GL-chitosan films with vanillin Schiff base and ZnO [36]. A similar trend was also observed in EB, ranging from 28.08% (GL/CMCS/CA-0%) to 33.24% (GL/CMCS/CA-3%). Alnadari et al. [37] demonstrated that the addition of *Cinnamomum camphora* fruit peel anthocyanins (ANC.P) significantly boosted the EB in sodium carboxymethyl cellulose-gum Arabic film from 45.38% to 65.39%. This consistency across different biopolymer systems and additive types indicates a potential common mechanism, possibly related to the interaction between polyphenolic compounds and the polymer chains, which may interfere with crystalline domain formation, improving film flexibility and durability. Hence, higher TS and EB values are desirable properties for packaging films, as they indicate a material’s ability to withstand mechanical stress during handling and transport, facilitate better sealing, and promote greater load capacity [38].

#### 3.1.3. MC, SD, and WS

The MC, SD, and WS of the films are quantified in Table 1. Moisture content is a critical parameter as it directly impacts the film’s physicochemical stability and potential for microbial growth. A lower MC is essential for maintaining the structural consistency and mechanical integrity of the film over time. The result showed a decrease in moisture content from 14.62% in GL/CMCS/CA-0% films to 12.41% in GL/CMCS/CA-3% films, consistent with the range (11.38 to 14.45%) exhibited in chitosan/GL/vanillin-Zn^2+^ films [36]. The decline is linked to the cross-linked network of CA, disrupting the access of water molecules to hydrophilic groups in the polymer matrix. For most biopolymer films, a common target for good mechanical and barrier stability is a moisture content below 20% as those beyond the limit often become overly soft, tacky, lose their mechanical integrity, and are susceptible to microbial growth.

SD and WS are critical indices that describe a film’s water retention properties. Excessive water solubility of a film compromises its structural integrity, leading to breakage during use. This breach triggers rapid quality deterioration through oxidation, microbial proliferation, and juice loss [8]. The result showed a marked decline in both parameters, ranging from 49.53 to 58.52% and 38.05 to 52.50%, respectively (*p* < 0.05), showcasing the impact of CA inclusion in promoting water resistance. The result corroborated the range of WS (41.23 to 58.13%) noted in quercetin-based chitosan-GL films [34]. The authors stated that the high water resistance of the films was attributed to the interaction of the amino group in GL and chitosan with the phenolic compound of quercetin.

#### 3.1.4. Color and Thickness

Table 2 highlights the color parameters (lightness = L*, redness = a*, and yellowness = b*), total color change (ΔE*), and thickness of the films. The color of the films plays a crucial role in the appearance of the coated food and consumer acceptance [26]. While CA addition into the GL/CMCS matrix did not influence the high L* values, as detected in GL/CMCS/CA-3% films (83.62 ± 0.19), significant changes were observed in the a*, b* and ΔE parameters. GL/CMCS/CA-3% films exhibited highest a* (1.38 ± 0.18) and ΔE* (12.84 ± 0.11), and the least b* (2.58 ± 1.06) values. Films with higher luminosity (L*) values are often preferred, as they denote a greater transparency that can enhance the acceptance of coated foods [39]. The decrease in a* and ΔE, and increase in b* values in CA-based films, are attributed to the intrinsic color of the compound, which is similar to the findings in previous studies [8,14].

Film thickness is a crucial feature that influences the barrier properties and mechanical strength needed for adequate packaging of foods. The incorporation of 3% CA significantly increased film thickness from 0.10 to 0.17 mm. This positive correlation between thickness and CA concentration underscores the direct influence of film composition. A similar thickness range (140.42 ± 1.05 to 148.10 ± 1.65 μm) was recently reported for GL-chitosan film loaded with *Nicotiana tabacum* extract nanoliposomes [40].

### 3.2. Film Characterization

#### 3.2.1. FTIR Spectra

FTIR spectroscopy provides valuable insights into the molecular arrangements and interactions between various components in composite films. When a sample is exposed to infrared radiation, it absorbs specific frequencies that excite its vibrational modes. These frequencies, measured in wavenumbers (typically 4000–400 cm^−1^), correspond to the stretching and bending of chemical bonds. The resulting absorption spectrum provides a unique fingerprint directly related to the molecule’s structure and bond properties [8].

**Figure 1:**
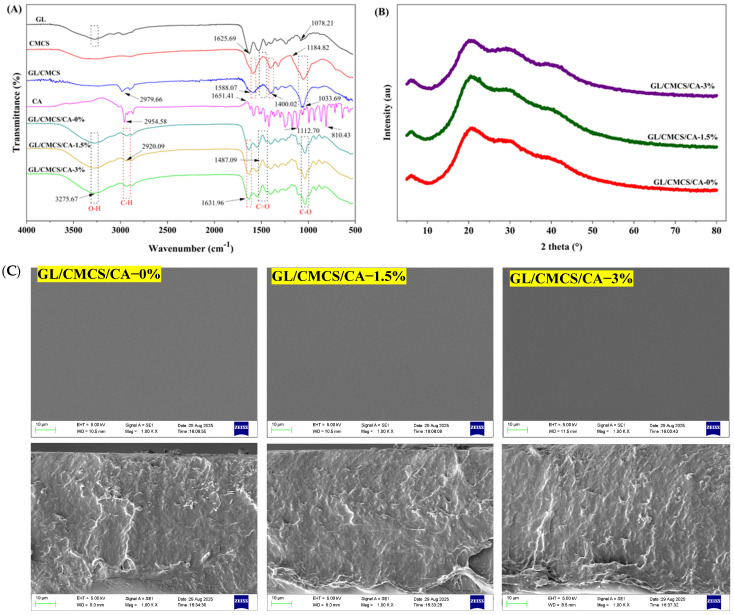
The FTIR (**A**), XRD (**B**), and SEM images (**C**) of the GL/CMCS/CA-based films.

As illustrated in Figure 1A, the dark dotted lines signify consistent peaks with the groups and the base materials (GL, CMCS, or CA), while the peaks highlighted in red represent similarities either with the groups or the base materials. Notably, characteristic absorption peaks of GL were detected at 1625.69 and 1078.21 cm^−1^, corresponding with the C=C stretch of alkene and C-O stretch of ether, respectively, consistent with 1634 cm-^1^ reported for GL [41]. The definite peaks of CMCS at 1588.07 and 1182.82 cm-^1^ are linked to the C=C stretch of the aromatic and C-N stretch of the amine groups, respectively. Ma et al. [35] detected similar peaks in CMCS at 1580, 1407, and 1178 cm^−1^, associated with the asymmetric stretching vibrations of -COOH and C-O-C, respectively. Additionally, the spectrum of the GL/CMCS blend exhibited characteristic peaks at 2979.66 cm^−1^ (C–H stretch of alcohol), 1588.07, and 1400.02 cm^−1^ (aromatic C=C stretch). The detection of the latter peaks in CMCS indicates its critical role in facilitating molecular interactions within the polymer network. In CA, distinct peaks were attributed to the C–H stretch of alcohol (2954.58 cm^−1^), C=C stretch of alkene (1651.41 cm^−1^), C-O stretch of ether (1112.70 cm^−1^), and =C–H bending of alkene (810.43 cm^−1^) [21].

In addition, notwithstanding the CA proportion, the characteristic peaks in the films were relatively similar, implying solution homogeneity and film stability. The primary peaks were synonymous with the O-H (H-bonded) (3275.67 cm^−1^) and C-H stretch (2920.09 cm^−1^) of the alcohol group, alkene C=C stretch (1631.96 cm^−1^), aromatic C=C stretch (1487.09 cm^−1^), and C–O stretch of ether (1033.69 cm^−1^), as also observed among the base materials. Biopolymers form stable films through hydrogen bonding with one another and/or with cross-linking agents. This is typical of the electrostatic and intermolecular hydrogen bonding between GL and CMCS. Our findings corroborate a previous study [42], which reported that a film’s overall strength and stability could be enhanced by secondary interactions, such as anthocyanin phenolic groups, hydrogen-bonding with the polymer matrix, and the formation of Schiff bases between compound C’s aldehyde CONnd polymer amines.

#### 3.2.2. XRD

XRD was employed to evaluate the CA’s effect on the crystallinity of the GL/CMCS-based films (Figure 1B). Crystallinity, which describes the degree of structural order within a solid, is a critical factor determining key material properties, including hardness, density, transparency, and diffusion. The result revealed a semi-crystalline nature of the film, characterized by a prominent peak at 2θ = 20.53°, showcasing the presence of amorphous phase structure. It also indicated that CA inclusion did not influence the crystalline structure of the matrix. Additional slight peaks (2θ = 6.35°, 30.06°, and 40.72°), especially the latter, became evident with increasing CA proportion, suggesting its role in creating a strong intra- and/or inter-molecular hydrogen bonding with the biopolymers, thereby improving polymer network, mechanical attributes, and bioactivity of the composite films. A recent study reported that as the sharper peak of chitosan in the films became broader, the diffraction shifted to a lower degree (18.85°), signifying the increase in the amorphous portion of the film and formation of longer chains during the film development process [5]. Although a similar finding was reported in GL/chitosan films loaded with *Cyclocarya paliurus* flavonoids [43], a contradicting phenomenon was observed in GL/chitosan films added with finger millet (*Eleusine coracana* L.), where the peak at 2θ became shorter with FMP inclusion [44].

#### 3.2.3. SEM

The surface and cross-sectional morphology of the GL/CMCS/CA films was characterized using SEM, a technique renowned for its high spatial resolution and ability to reveal topographic and morphological features (Figure 1C). This technique was critical for evaluating the homogeneity and compatibility of the blended materials. Observed defects in the film, including pores, surface coarseness, and fractures, are directly correlated with inferior mechanical strength, making it unsuitable for food packaging. Although the films exhibited marginally coarser surface characteristics with increasing CA concentration, they maintained a relatively uniform and compact morphology. A similar result was observed in chitosan/GL/salicylic acid (SA) films, where SA addition increased roughness [5]. The increase in surface roughness significantly enhances the film’s effectiveness in preserving food quality. This textural modification enhances the adhesion of the film to food surfaces by augmenting the interfacial contact area for bonding. The resultant improvement in mechanical durability strengthens the coating’s barrier properties, offering more robust protection against moisture, oxidative reactions, and physical damage [45].

Moreso, the cross-sectional images confirmed the structural integrity of the films, signifying an excellent compatibility and effective interactions between CA and the biopolymers. The consistency of this morphology with fish scale-derived GL/sodium alginate/CA-loaded ZIF-8 films [46] underscores CA’s role as a compatible crosslinking agent in the film-forming process.

### 3.3. Physicochemical Analyses of the Strawberries

#### 3.3.1. WL and Decay Rate

**Figure 2:**
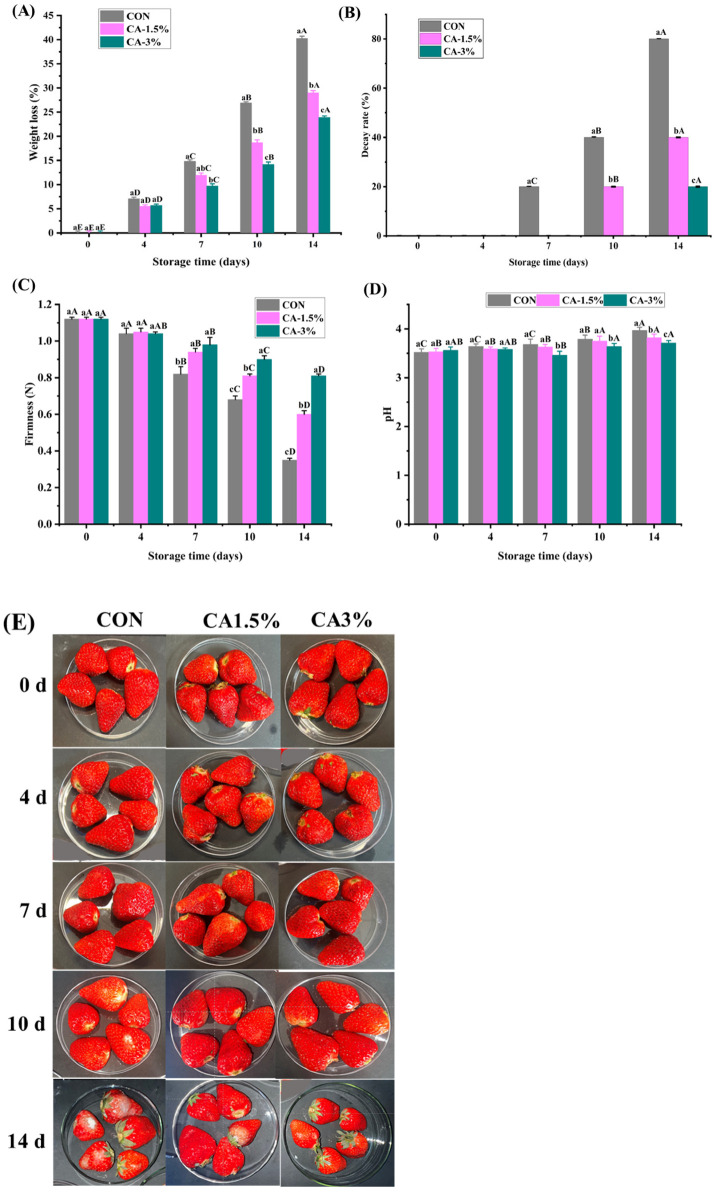
The WL (**A**), decay rate (**B**), firmness (**C**), pH (**D**), and pictorial description (**E**) of uncoated and GL/CMCS/CA-coated strawberries at 4 °C storage for 14 days. Different lowercase letters (a–c) indicate significant variations between treatment groups within a storage day (*p* < 0.05), while different uppercase letters (A–E) indicate significant variation across storage days within a treatment group. Error bars represent the standard deviation of replicates (n = 3).

Water release from fruits into the environment increases during storage. Hence, measuring WL serves as an indicator of fruit freshness. As presented in Figure 2A, the WL steadily increased as storage progressed (*p* < 0.05), ranging from 23.93% (CA-3%) to 40.28% (CON) at the end of storage (day 14). Coating acts as a barrier that effectively restricts water movement and fruit transpiration, which are primarily causes of water loss in fruits [47]. The result was lower than the range (31.89 ± 10.25–43.09 ± 13.58%) reported for uncoated and chitosan/gelatin/pomegranate peel extract (CGPPE)-coated strawberries after 12 days storage at 7 °C [3]. Nonetheless, the values were higher than 11.1–15.6% demonstrated in untreated and chitosan-treated strawberries stored at 4 °C for 21 days [48]. The authors posited that chitosan coating reduced WL by forming a thin, semi-permeable layer on the fruit surface, while the higher WL in untreated fruits was due to exposed surface pores. The result confirms that treatment and storage time can affect WL reduction, and the effects of polymeric substances are not source-dependent.

Strawberry spoilage is often caused by microbial invasion and mechanical damage. No decay was detected on days 0 and 4, affirming the freshness of the berries (Figure 2B). As storage time progressed, the CON samples exhibited the highest rate, with most strawberries being shriveled or infested with microorganisms. Guo et al. [49] also reported variations in fungal decay percentages of about 75% in the control group during 8 days of storage. In contrast, the coated strawberries were intact and rosy, with only one showing decay in CA-3% after 14 days. The result differed from that of Pinzon et al. [50] and Bertolo et al. [3], which showed an exceedance of 60% decay threshold in strawberries coated with banana starch–chitosan–aloe vera gel and CGPPE after 15 and 12 days of refrigerated storage, respectively. This significant distinction can be attributed to the combined efficacy of CA and the intrinsic antimicrobial activity of CMCS, which together create a physical barrier that inhibits microbial growth on the fruit surface.

#### 3.3.2. Firmness and pH

The firmness of strawberries not only indicates freshness and customer acceptability but also signifies the degradation of structural compounds due to their soft texture and fragile skin. As highlighted in Figure 2C, the initial value was 1.12 N. The firmness decreased in the groups during storage, with no significant change detected on days 0 and 4 (*p* > 0.05). As storage progressed, a marked downtrend was noted in control compared to the coated groups from day 7, with values ranging from 0.35 to 0.81 N on day 14 between CON and CA-3%, respectively (*p* < 0.05). The loss of firmness (softening) in strawberries is primarily attributed to cell wall degradation. This process is accelerated by microbial activity (especially fungi), fruit respiration, and moisture loss [51]. Interestingly, GL, CMCS, and CA retained fruit firmness, which validates the potency of their carrier-forming properties in preventing fungal contamination and structural changes during storage (Figure 2D). Several studies have also demonstrated better firmness retention in coated strawberries than in uncoated ones, regardless of the formulation used in previous studies [3,4,5,52], which corroborates our findings.

The level of acidity in fruits is a crucial indicator of quality and consumer acceptability. Strawberries exhibit an acidic pH range of 3.0 to 3.5. This acidity naturally preserves the fruit and is responsible for its signature tart taste [5]. As presented in Figure 2D, the initial average pH value of the groups was 3.54 ± 0.11, which is within the usual pH range. Nevertheless, there was a gradual increase as storage progressed, suggesting a continuous maturation of the strawberries during storage. The values of the CON strawberries increased by 12.78% (3.97) compared to 4.21% (3.71) in the coated group (CA-3%) on day 14. The measured pH was slightly elevated compared to the range of 3.3–3.53 documented for strawberries coated with chitosan and *M. spicata* essential oil [53], but within the range (3.3–3.75) noted in CGPPE-coated strawberries [3]. The significant distinction could be attributed to the inhibition of organic acid degradation by CA, which distorted respiratory metabolism in coated samples during storage. Maintaining a more acidic pH in coated fruits is also essential, as it guarantees an effective control of microbial growth [11].

#### 3.3.3. Color

**Figure 3:**
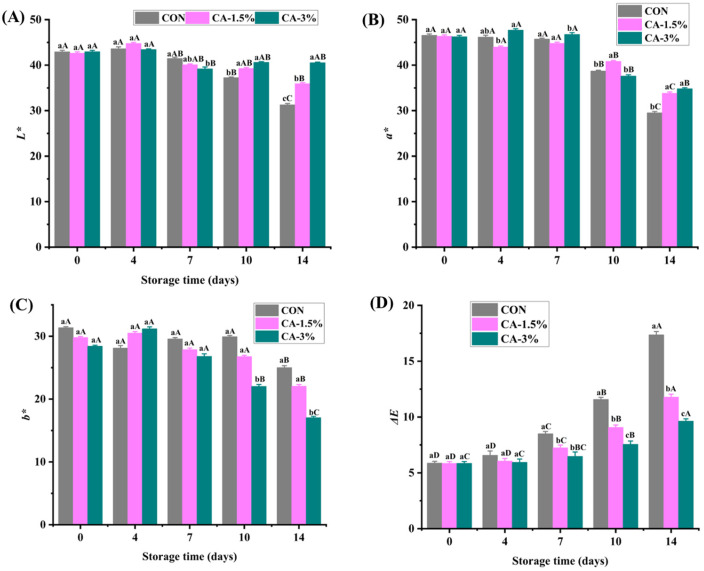
The color properties: L* (**A**), a* (**B**), b* (**C**), and Δ*E* (**D**) of uncoated and GL/CMCS/CA-coated strawberries at 4 °C storage for 14 days. Different lowercase letters (a–c) indicate significant variations between treatment groups within a storage day (*p* < 0.05), while different uppercase letters (A–D) indicate significant variation across storage days within a treatment group. Error bars represent the standard deviation of replicates (n = 3).

Color is a crucial characteristic that consumers use when purchasing fruits and vegetables; therefore, the need for its evaluation during strawberry storage was evident in this study (Figure 3A). As illustrated in Figure 2A, the mean L* content of the samples was 42.72 on day 0. The L* signifies the lightness of a sample and ranges from 0 (pure black) to 100 (pure white). No significant change was detected until day 7 (*p* > 0.05), after which a gradual decline was observed throughout storage, ranging between 31.28 (CON) and 40.52 (CA-3%). The variation can be attributed to the moisture loss on the surface of the fruits, which accelerated the ripening process, consequently diminishing their luminosity [28]. Brown stains and black spots, caused by fungal growth or improper storage conditions, can also exacerbate the decline of L* content [54]. Conversely, the a* content in the groups slightly increased till day 7, followed by a decrease throughout storage (Figure 3B). Maintaining a* content (redness) is associated with the proportion of anthocyanins in fruits [55]. The observed discoloration can be attributed to the enzymatic degradation of anthocyanin pigments. Specifically, hydrolytic enzymes present in the fruit tissue target and break the critical glycosidic linkages that stabilize these compounds, resulting in the irreversible loss of color during storage [54]. The b* content exhibited a similar trend, with a sharp increase observed in the coated group on day 4, followed by a gradual decline (*p* < 0.05), with the lowest value (17.04) noted in CA-3% at the end of storage (Figure 3C). Another study also demonstrated a similar trend in the b* content of coated strawberries during 14-day storage at 3 ± 1 °C [5].

Furthermore, the initial Δ*E* was 5.86 in the groups. However, a significant variation was detected in CON from day 7 (8.51), reaching 17.36 on day 14 compared to 9.63 exhibited in CA-3%-coated strawberries (*p* < 0.05). The finding corroborates the higher a* values observed in CA-3% samples. The increase can be attributed to the rapid exposure of the uncoated strawberries to the oxidative and dehydration processes during storage [50]. Edible coatings can alter physiological actions in the fruit, suppressing respiratory activity and transpiration rates, and inhibiting microbial contamination on the fruit surface, thereby delaying pigment transformation during ripening [56].

### 3.4. Biochemical and Antioxidant Activities of Coated Strawberries

#### 3.4.1. Total Soluble Solids (TSS) and Titratable Acidity (TA)

**Figure 4:**
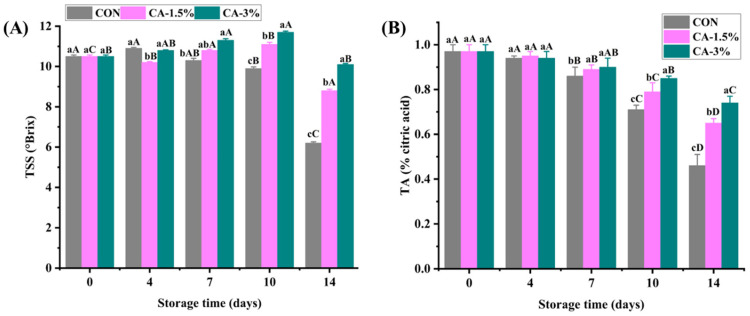
The TSS (**A**) and TA (**B**) of uncoated and GL/CMCS/CA-coated strawberries at 4 °C storage for 14 days. Different lowercase letters (a–c) indicate significant variations between treatment groups within a storage day (*p* < 0.05), while different uppercase letters (A–D) indicate significant variation across storage days within a treatment group. Error bars represent the standard deviation of replicates (n = 3).

TSS and TA are essential indices for evaluating strawberry flavor. The initial TSS was 10.50 °Brix in the groups during storage (Figure 4A). The values gradually increased in the coated strawberries compared to the control, peaking at 11.70 °Brix in CA-3% on day 10. The TSS decreased to 6.20 °Brix (CON) and 10.10 °Brix (CA-3%) on day 14. A similar trend was observed in strawberries coated with chitosan/pullulan/thyme essential oil under 2 °C for 9 days, which was substantially higher compared to other groups (*p* < 0.05), suggesting that the coating materials stabilized the flavor of the fruits [4]. Although the TSS of strawberries often declines with increasing storage as a result of continuous metabolism, including glucose production for fruit respiration [52], other authors posited that a rise could occur during storage due to starch to sugar conversion, polysaccharide hydrolysis in the cell wall, and increased sugar concentrations from water loss in the fruits [57,58].

In strawberries, citric acid is the primary acid and therefore the one predominantly measured by TA. As illustrated in Figure 4B, TA (% citric acid) gradually decreased in the strawberries as storage progressed, with a marked variation noted from day 7 throughout storage. The values ranged from 0.46 to 0.74% citric acid in Groups A and C, respectively, indicating the effectiveness of GL/CMCS/CA coating in delaying TA decline during postharvest storage. Additionally, the CA exhibits antibacterial attributes that can suppress microbial proliferation on the fruit surface, subsequently mitigating spoilage and ripening during storage. The uncoated strawberries in a previous study also showed the fastest TA decline compared to gum Arabic/guar gum-coated ones after storage for 16 days at 4 °C, which could be linked to the higher respiration rate and rapid utilization of organic acids during storage [59]. Moreover, coatings disrupt the overall respiratory metabolism in fruits, which in turn alters the citric acid cycle and organic acid synthesis, maintaining TA in the process [60].

#### 3.4.2. TPC

**Figure 5:**
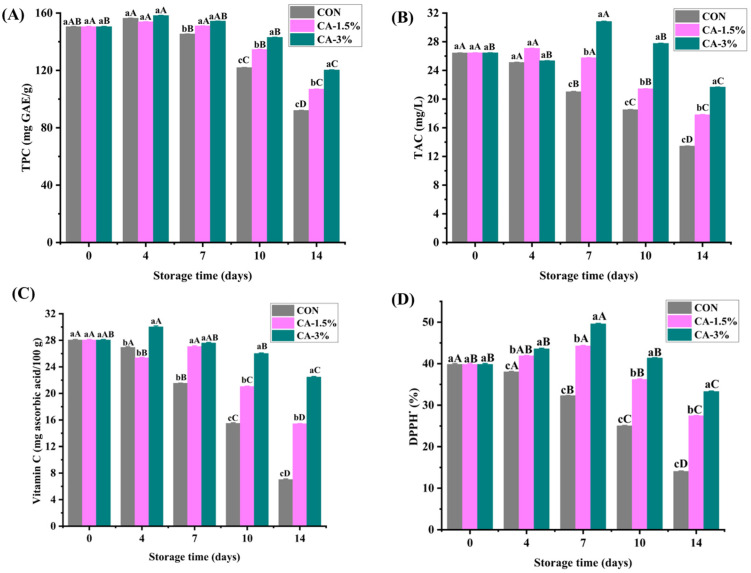
The TPC (**A**), TAC (**B**), vitamin C (**C**), and antioxidant activity (**D**) of uncoated and GL/CMCS/CA-coated strawberries at 4 °C storage for 14 days. Different lowercase letters (a–c) indicate significant variations between treatment groups within a storage day (*p* < 0.05), while different uppercase letters (A–D) indicate significant variation across storage days within a treatment group. Error bars represent the standard deviation of replicates (n = 3).

Figure 5A illustrates the TPC (mg GAE/g strawberry) of the strawberries during storage. The initial TPC was 150.48 mg GAE/g in the groups. In CON, a slight increase was observed on day 4, followed by a significant decrease to 92.06 mg GAE/g on day 14, which may be attributed to the rapid metabolism of the uncoated strawberries. However, in the coated group (CA-3%), a significant increase was observed on day 4 (158.22 mg GAE/g), followed by a slight decrease on day 7 (154.37 mg GAE/g) and then a gradual decline to the last day (120.27 mg GAE/g). Zebua et al. reported a similar result for pectin-coated strawberries loaded with lemon peel extract on day 8, with the coating materials inducing an increase in TPC under cold storage (4 °C) for 12 days [61]. Changes in TPC during storage may vary depending on the fruit’s characteristics, such as its degree of ripeness and the type of coating.

#### 3.4.3. TAC

As demonstrated in Figure 5B, the initial TAC in the groups was 26.45 mg/L. While the content gradually decreased in CON throughout storage, reaching 13.45 mg/L on the last day, the coated strawberries exhibited a sharp increase on day 7 (30.82 mg/L), followed by a decrease to 21.67 mg/L on day 14 (*p* < 0.05). Coatings could promote this phenomenon in fruits by changing their internal properties and disrupting the biochemical reactions that result in the synthesis of anthocyanins. This helps to maintain the redness of the fruits during storage. The result aligned with a previous study that showed a TAC increase on day 8, before declining throughout storage [61], corroborating the assertion that TAC decrease could arise from disturbances and senescence in the fruits that lead to the cessation of anthocyanin biosynthesis [60].

#### 3.4.4. Vitamin C

The Vitamin C content in the coated and uncoated strawberries is presented in Figure 5C. The content was consistent in the groups (28.05 mg ascorbic acid/100 g) on day 0. As the storage time progressed, the content in CON kept decreasing throughout storage, reaching 7.02 mg ascorbic acid/100 g on day 14. In CA-3% samples, however, an increase (30.04 mg ascorbic acid/100 g) was noted on day 4, followed by a gradual decline to 22.47 mg ascorbic acid/100 g at the end of storage. Previous studies also reported a similar trend in the content of coated strawberries during refrigerated storage [3,28]. Vitamin C (ascorbic acid) is susceptible to oxidation and can be lost through moisture leakage, resulting in degradation over time. GL/CMCS/CA coating was beneficial in forming a semi-permeable barrier around the fruits, which helped modify the internal atmosphere (lower O_2_ and higher CO_2_). Consequently, this slowed down respiration, enzymatic activity, and oxidative reactions associated with ripening during storage. Oliveira Filho et al. posited that the irreversible oxidation of ascorbic acid to dehydroascorbic acid, catalyzed by atmospheric oxygen, may cause Vitamin C degradation [28].

#### 3.4.5. Antioxidant Activity

Antioxidant activity is a crucial measure of the effectiveness of substances in scavenging free radicals in a medium. Adding antioxidants to the coating can enhance performance against oxidative reactions and microbial growth, while preserving organoleptic traits and prolonging the shelf life of foods. The antioxidant activity of the coating, determined using the DPPH method, is presented in Figure 5D. The result showed a DPPH of 72.79% in the groups on day 0, which was significantly higher than the ~50% DPPH scavenging activity reported in strawberries coated with chitosan/water caltrop pericarp extract [62] and lower than 81.10% DPPH in strawberries coated with xanthan gum with sodium nitroprusside [63]. As days progressed, the DPPH activity peaked on day 7 (79.56%) in CA-3% but gradually declined in CON until the end of storage (35.19%). The decline over time could be attributed to the synthesis of antioxidant compounds, including anthocyanins and phenolics, which temporarily enhance the fruit’s antioxidant activity [64]. The CA addition in the coating solution promoted antioxidant activity, with its capability to scavenge DPPH free radicals increasing significantly with the proportion. Interestingly, the CA’s antioxidant property involves its strong hydrogen-donating ability to reduce the activity of radicals or eliminate them [65].

### 3.5. Microbiological Assessment

**Figure 6:**
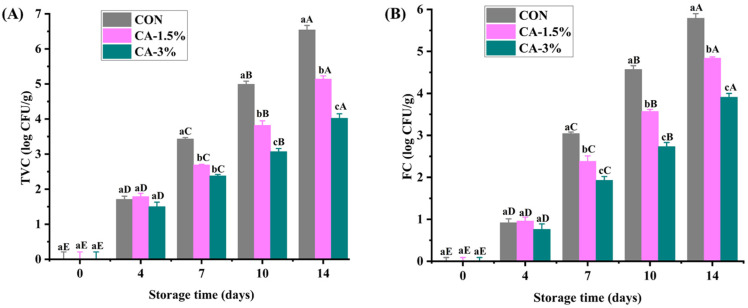
The TVC (**A**) and FC (**B**) of uncoated and GL/CMCS/CA-coated strawberries at 4 °C storage for 14 days. Different lowercase letters (a–c) indicate significant variations between treatment groups within a storage day (*p* < 0.05), while different uppercase letters (A–E) indicate significant variation across storage days within a treatment group. Error bars represent the standard deviation of replicates (n = 3).

The rich nutritional composition of strawberries exposes them to microbial contamination, making microbiological assessment critical for verifying freshness and controlling the economic losses associated with postharvest quality decline. Figure 6A highlights the TVC in the coated and uncoated strawberries. The result showed no bacterial contamination on day 0, affirming the freshness of the strawberries. However, growth was detected from day 4 throughout storage, reaching 6.54, 5.14, and 4.02 log CFU/g on day 14 in CON, CA-1.5%, and CA-3% groups, respectively (*p* < 0.05). The values were considerably lower than the 7.2 log CFU/g and 5.63 log CFU/g reported for control and Xyloglucan–Borassus flabellifer seed-coated strawberries after 8 days of storage [66].

Similarly, FC showed no detectable levels on day 0 (Figure 6B), compared to the >3 log CFU/g reported in strawberries with CMC-*Lactobacillus plantarum* edible coating [1]. The growth was also negligible on day 4, indicating insignificant fungal growth during the initial storage period. Although the load in CON increased rapidly from day 7, reaching 5.79 log CFU/g on the last day, compared to 3.91 log CFU/g observed in CA-3% group, it remained within the permissible limit of 6 log CFU/g established by the Institute of Food Science and Technology (IFST, London, UK) [1]. The antimicrobial properties of CMCS and CA resulted in the lowest counts of microorganisms in the coated groups, tantamount to their effectiveness in maintaining strawberry quality and enhancing their shelf life without synthetic substances.

### 3.6. Sensory Evaluation

Sensory evaluation is a critical tool for assessing postharvest fruit quality, as it directly measures the attributes that most influence consumer preference. The traits (appearance, firmness, taste, and overall acceptance) were assessed by 12 panelists using a 9-point Hedonic scale, and the mean values are presented (Table 3). Quality parameters remained stable from day 0 to day 4, indicating no initial deterioration in strawberry freshness. Given that sensory attributes are inherently linked to fruit freshness, Bertolo et al. [3] observed no change in the initial sensory profiles of CGPPE-coated and uncoated strawberries, corroborating our result. As storage progressed, the uncoated (CON) strawberries exhibited a significant downtrend in the sensory scores from day 7 in appearance (7.92), firmness (7.25), taste (7.92), and overall acceptance (7.33) compared to 8.83, 8.67, 8.67, and 8.67 scores in CA-3%-coated ones, respectively. On day 14, the scores ranged from 3.75 to 6.91, 3.58 to 6.42, 3.67 to 5.16, and 3.83 to 6.17, indicating a 45.73, 44.24, 28.88, and 37.93% increase, respectively, in CA-3%-coated strawberries at the end of storage (*p* < 0.05). This sharp decline in the CON group inferred substantial quality changes, attributable to enzymatic degradation, oxidative processes, and microbial growth. A similar finding was reported in a recent study, where the retention of sensory characteristics was attributed to the antimicrobial properties of savory essential oil (SEO), which likely inhibited microbial growth and preserved the volatile aromatic compounds in nano-SEO-coated strawberries [31].

## 4. Conclusions

Based on the comprehensive evaluation of this study, GL/CMCS-based coatings, functionalized with CA, demonstrated significant potential as effective, bio-based alternatives to synthetic preservatives for extending strawberry shelf life. The incorporation of CA, particularly at 3%, markedly improved the films’ physical, mechanical, and barrier properties while ensuring homogeneous integration within the polymer matrix. During storage, strawberries coated with GL/CMCS/CA-3% exhibited superior quality retention, reduced weight loss and fungal decay, and maintained firmness, color, and pH. The coating also effectively improved the antioxidant capacity, suppressed microbial proliferation, and retained sensory profiles in the samples. These findings confirm that CA-enriched GL/CMCS coatings can preserve postharvest strawberry quality and prolong shelf life through multifunctional mechanisms, offering a promising natural solution for sustainable food preservation.

## Figures and Tables

**Table 1:** Physico-mechanical characteristics of GL/CMCS/CA-based films.

Films	Opacity (mm)	WVP (×10^−11^ g^−1^ s^−1^ Pa^−1^)	TS (MPa)	EB (%)	MC (%)	SD (%)	WS
GL/CMCS/CA-0%	1.64 ± 0.13 ^c^	4.07 ± 0.11 ^a^	28.38 ± 0.20 ^c^	28.08 ± 0.09 ^c^	14.62 ± 0.10 ^a^	58.52 ± 0.11 ^a^	52.50 ± 0.18 ^a^
GL/CMCS/CA-1.5%	3.15 ± 0.17 ^b^	2.84 ± 0.07 ^b^	36.52 ± 0.05 ^b^	31.26 ± 0.11 ^b^	13.03 ± 0.03 ^b^	55.27 ± 0.08 ^b^	42.57 ± 0.10 ^b^
GL/CMCS/CA-3%	3.74 ± 0.31 ^a^	2.51 ± 0.08 ^c^	42.70 ± 0.08 ^a^	33.04 ± 0.05 ^a^	12.41 ± 0.07 ^c^	49.53 ± 0.09 ^c^	38.05 ± 0.07 ^c^

EB = elongation-at-break; MC = moisture content; SD = swelling degree; TS = tensile strength; WS = water solubility; WVP = water vapor permeability. Significant variations among the films (*p* < 0.05) are denoted by the superscripts (a–c).

**Table 2:** Color and thickness of GL/CMCS/CA-based films.

Films	L*	a*	b*	ΔE*	Thickness (mm)
GL/CMCS/CA-0%	82.19 ± 0.06 ^a^	1.38 ± 0.18 ^a^	2.58 ± 1.06 ^c^	12.84 ± 0.11 ^a^	0.10 ± 0.07 ^c^
GL/CMCS/CA-1.5%	83.71 ± 0.11 ^a^	1.35 ± 1.03 ^ab^	3.27 ± 0.50 ^b^	11.05 ± 0.38 ^b^	0.15 ± 0.06 ^b^
GL/CMCS/CA-3%	83.62 ± 0.19 ^a^	1.33 ± 0.37 ^b^	3.58 ± 0.51 ^a^	10.24 ± 0.09 ^b^	0.17 ± 0.09 ^a^

Values are expressed as means ± SD (n = 3). Significant variations among the films (*p* < 0.05) are denoted by the superscripts (a–c).

**Table 3:** Sensory evaluation of uncoated and GL/CMCS/CA-coated at 4 °C storage for 14 days. The superscripts (a–c) indicate significant variations between treatment groups within a storage day (*p* < 0.05), while (A–D) indicate significant variation across storage days within a treatment group. Values are presented as mean + standard deviation of replicates (n = 3).

Storage Time (Days)	Group	Sensory Characteristics			
		Appearance	Firmness	Taste	Overall Acceptance
**0**	CON	9.00 ± 0.00 ^aA^	9.00 ± 0.00 ^aA^	9.00 ± 0.00 ^aA^	9.00 ± 0.00 ^aA^
	GL/CMCS/CA-1.5%	9.00 ± 0.00 ^aA^	9.00 ± 0.00 ^aA^	8.92 ± 0.16 ^aA^	8.83 ± 0.09 ^aA^
	GL/CMCS/CA-3%	9.00 ± 0.00 ^aA^	9.00 ± 0.00 ^aA^	9.00 ± 0.00 ^aA^	9.00 ± 0.00 ^aA^
**4**	CON	8.75 ± 0.18 ^aA^	8.92 ± 0.16 ^aA^	8.92 ± 0.16 ^aA^	8.92 ± 0.16 ^aA^
	GL/CMCS/CA-1.5%	8.75 ± 0.18 ^aAB^	8.92 ± 0.16 ^aA^	8.83 ± 0.09 ^aA^	8.75 ± 0.18 ^aA^
	GL/CMCS/CA-3%	8.83 ± 0.09 ^aA^	8.92 ± 0.16 ^aA^	8.92 ± 0.16 ^aA^	8.83 ± 0.09 ^aA^
**7**	CON	7.92 ± 0.14 ^bB^	7.25 ± 0.28 ^bB^	7.92 ± 0.14 ^aB^	7.33 ± 0.10 ^cB^
	GL/CMCS/CA-1.5%	8.25 ± 0.09 ^aB^	8.25 ± 0.09 ^aB^	8.00 ± 0.10 ^abB^	8.25 ± 0.09 ^bB^
	GL/CMCS/CA-3%	8.83 ± 0.09 ^aA^	8.67 ± 0.12 ^aA^	8.67 ± 0.12 ^bA^	8.67 ± 0.12 ^aA^
**10**	CON	6.58 ± 0.18 ^cC^	5.92 ± 0.15 ^cC^	6.17 ± 0.12 ^bC^	6.17 ± 0.12 ^cC^
	GL/CMCS/CA-1.5%	7.17 ± 0.36 ^bC^	6.58 ± 0.18 ^bC^	5.83 ± 0.20 ^aC^	6.67 ± 0.19 ^bC^
	GL/CMCS/CA-3%	7.50 ± 0.11 ^aB^	7.17 ± 0.36 ^aB^	5.83 ± 0.20 ^aB^	6.91 ± 0.08 ^aB^
**14**	CON	3.75 ± 0.07 ^cD^	3.58 ± 0.30 ^cD^	3.67 ± 0.32 ^cD^	3.83 ± 0.16 ^cD^
	GL/CMCS/CA-1.5%	4.58 ± 0.24 ^bD^	5.16 ± 0.15 ^bD^	4.33 ± 0.27 ^bD^	4.58 ± 0.24 ^bD^
	GL/CMCS/CA-3%	6.91 ± 0.08 ^aC^	6.42 ± 0.20 ^aC^	5.16 ± 0.15 ^aC^	6.17 ± 0.12 ^aC^

## Data Availability

The original contributions presented in the study are included in the article. Further inquiries can be directed to the corresponding author.
